# Replica symmetry breaking in 1D Rayleigh scattering system: theory and validations

**DOI:** 10.1038/s41377-024-01475-5

**Published:** 2024-07-02

**Authors:** Yifei Qi, Longqun Ni, Zhenyu Ye, Jiaojiao Zhang, Xingyu Bao, Pan Wang, Yunjiang Rao, Ernesto P. Raposo, Anderson S. L. Gomes, Zinan Wang

**Affiliations:** 1 Key Lab of Optical Fiber Sensing & Communications, University of Electronic Science and Technology of China (UESTC), Chengdu, China; 2 Laboratório de Física Teórica e Computacional, Departamento de Física, Universidade Federal de Pernambuco, 50670-901 Recife, Pernambuco Brazil; 3 Departamento de Física, Universidade Federal de Pernambuco, Recife, Pernambuco Brazil

**Keywords:** Nonlinear optics, Magneto-optics

## Abstract

Spin glass theory, as a paradigm for describing disordered magnetic systems, constitutes a prominent subject of study within statistical physics. Replica symmetry breaking (RSB), as one of the pivotal concepts for the understanding of spin glass theory, means that under identical conditions, disordered systems can yield distinct states with nontrivial correlations. Random fiber laser (RFL) based on Rayleigh scattering (RS) is a complex disordered system, owing to the disorder and stochasticity of RS. In this work, for the first time, a precise theoretical model is elaborated for studying the photonic phase transition via the platform of RS-based RFL, in which we clearly reveal that, apart from the pump power, the photon phase variation in RFL is also an analogy to the temperature term in spin-glass phase transition, leading to a novel insight into the intrinsic mechanisms of photonic phase transition. In addition, based on this model and real-time high-fidelity detection spectral evolution, we theoretically predict and experimentally observe the mode-asymmetric characteristics of photonic phase transition in RS-based RFL. This finding contributes to a deeper understanding of the photonic RSB regime and the dynamics of RS-based RFL.

## Abstract (web summary)

This work reveals intrinsic mechanisms of photonic phase transitions with a precise theoretical model in 1D Rayleigh scattering system, and could have broad and profound impacts across various complex systems.

## Graphical abstract

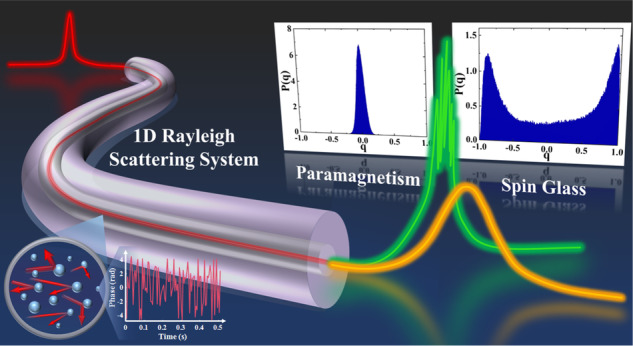

## Introduction

The spin glass theory gained widespread attention since the 1970s, serving as a framework to describe a critical state in disordered magnetic systems in which nontrivially correlated spins “freeze” at random directions below some critical temperature^1–4^. Subsequently, Sherrington and Kirkpatrick made significant contributions by proposing a mathematical model based on Ising model, where spins are coupled by an infinite range of random interactions^5^, thereby conducting an in-depth exploration of the spin glass theory. To provide a more comprehensive description of complex phenomena within spin glass systems and circumvent technical difficulties related to the lack of stability of the Sherrington and Kirkpatrick solution, Parisi introduced the Replica symmetry breaking (RSB) theory^6,7^. When a system is in a spin glass state, it exhibits numerous local minima associated with configuration states in the free energy landscape. In this regime, identical replicas under the same experimental condition can manifest distinct properties, a phenomenon known as RSB, effectively characterized using the Parisi overlap parameter^8^. Considering the importance of RSB not only for statistical physics, but also its connection to turbulence behavior^9^, the 2021 Nobel Prize in Physics was partially laureated to Giorgio Parisi “for the discovery of the interplay of disorder and fluctuations in physical systems from atomic to planetary scales”. Research into the spin glass theory contributes to the understanding of the properties of complex systems characterized by non-uniform and disordered attributes, and extends its applicability to fields such as materials science, neural networks, and condensed matter physics^10–13^.

Recently, the RSB spin-glass theory has been applied to investigate various disordered systems, including random lasers and nonlinear wave propagation^14–16^. In 2015, Ghofraniha et al. made a significant breakthrough by observing the RSB phenomenon for the first time in experiments using a solid-state random laser platform. They inferred the Parisi overlap parameter from fluctuations in spectral intensity, and the experiments revealed that, as the pump power transitions from below the threshold to above the threshold, the random laser undergoes a phase transition from a photonic paramagnetic state to a spin glass state^17^. In 2016, Gomes et al. demonstrated simultaneous observation of RSB and Lévy behavior in an Nd-powder cased random laser (RL)^18^, and Pincheira et al. reported the occurrence of RSB in a colloidal-based RL^19^, in agreement with theoretical predictions^20,21^. In the same year, Tommasi et al. conducted experiments confirming the RSB phase transition phenomenon in different disordered random lasers^22^. In 2017, Pierangeli et al. observed the RSB phenomenon in the context of nonlinear wave propagation^23^.

The experiments mentioned above have predominantly utilized complex three-dimensional waveguide materials, which are sensitive to environmental factors and present challenges in generating replicas. In contrast, random fiber laser (RFL), as an important subclass of random laser, provides a one-dimensional platform for studying spin glass theory, which encapsulates disorder in feedback and gain within a one-dimensional optical fiber waveguide^24,25^, and a review of the RFL research related to the 2021 Nobel Prize in Physics is reported^26^. The core idea for investigating the spin glass theory in photonic systems, such as random lasers, is the analogy of the lasing modes to spin variables^27^. In addition, random lasers, akin to an inverse-temperature system, exhibit different laser states under varying pump energies, transitioning from a photonic paramagnetic phase at low pump energies to a spin glass phase at high pump energies. Gomes et al. first observed RSB in erbium-doped fiber random lasers, introducing disorder through a random fiber grating^28,29^. Compared with the random fiber grating, the Rayleigh scattering (RS) in optical fibers has a higher degree of disorder, making the RS-based RFL a natural ideal platform for the study of photon phase transition. Moreover, due to the advantages of high efficiency^30^, good beam quality^31^, wavelength agility^32^, and low noise^33^, RS-based RFL^34^ not only have wide-range applications in remote sensing^35^, distributed amplification^36^, imaging^37^ and high-power lasers^38^, but also are currently considered as one of the seed source candidates for next-generation laser inertial confinement fusion devices^39^. Therefore, proposing a perfect simulation model to study the intrinsic photon phase transition mechanism, and accurately grasp its working state, is crucial for its application in various fields. However, the current experiments tend to require long time intervals for replica acquisition and lack a corresponding numerical simulation platform to fill the theoretical gap.

In this work, the intrinsic mechanisms of photonic phase transition in an RS-based RFL system are investigated through both theoretical and experimental approaches for the first time, to the best of our knowledge. Benefiting from the precise calibration of RS phase fluctuations over time with unprecedented precision, an RS-phase-variation model of RS-based RFL is proposed, which is well validated by high-precision spectral measurements. Then, based on this model and real-time detection of high-fidelity spectral evolution, we find not only that the photon phase variation in RS-based RFL can be analogous to the temperature in the phase transition but also that the photonic phase transition in RS-based RFL exhibits mode-asymmetry. This finding not only provides a novel experimental and simulation platform for RSB research but also advances our understanding of complex systems.

## Results

### Theoretical analysis

As a start, a numerical analysis is carried out to study the dynamic characteristics of the RS-based RFL. The simulation model is based on the generalized nonlinear Schrödinger equations (NLSEs)^40,41^. Details of this model are given in “Materials and methods”.

**Fig. 1:**
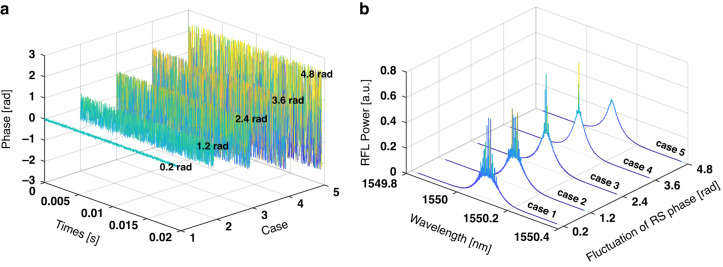
The RS phase with different fluctuation ranges and the corresponding spectrum. **a** Five different fluctuation ranges of RS phase in simulation: Case 1: 0.2 rad, Case 2: 1.2 rad, Case 3: 2.4 rad, Case 4: 3.6 rad, Case 5: 4.8 rad. **b** Corresponding evolution of spectrum: as the degree of RS phase fluctuation increases, the randomly distributed spikes of RFL spectrum gradually disappear

It should be noted that, as a pivotal parameter in RS-based RFL, the RS phase does not remain constant, but changes according to the external environment, and the degree of RS phase fluctuation will determine the excited state of RS-based RFL. Thus, the RS-based RFL with different fluctuating states of RS phase is simulated. As shown in Fig. 1a, the fluctuation of RS phase changed from 0.1 rad to 4.8 rad, and the corresponding RS-based RFL output characteristics are shown in Fig. 1b. The simulation results show that, when the fluctuation of RS phase is small, such as 0.1 rad to 3.6 rad, the spectrum of the RFL exhibits randomly distributed spikes, and the number of the spikes decreases as the degree of RS-phase fluctuation increases; conversely, when the fluctuation of RS phase is large, such as 4.8 rad, the RFL has a smooth spectrum. The reason for this is as follows. The RS reflection spectrum has random distributed spikes^42^ (as shown in Fig. S2), and when the fluctuation of the RS phase is small, the RS reflection spectrum can remain stable (as shown in Fig. S3a). Therefore, a stable, coherent resonant cavity can be formed, and random longitudinal modes are excited, which are manifested in the spectrum as randomly distributed spikes. However, when there is an external action causing large fluctuations in the RS phase, the reflection spectrum of the RS undergoes a dramatic change (as shown in Fig. S3b). Consequently, it fails to establish stable feedback, indicating that the random longitudinal modes cannot be established.

The above results show that the degree of the RS phase fluctuation determines the excited state of RS-based RFL. Therefore, determining the degree of the fluctuations of the RS phase in the experimental environment is particularly important for accurately simulating the excitation state of the RS-based RFL, and it was measured by a $\Phi$-OTDR with proprietary technologies^43^ (details shown in Supplementary Information). This refinement process has contributed to the attainment of a high-fidelity representation of RS-based RFL behavior within the simulations.

### Experimental validation of theoretical model

**Fig. 2:**
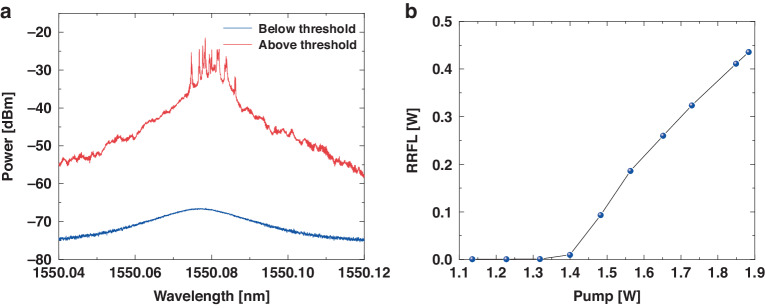
The spectrum of RFL at different pump power and RFL output power curve. **a** The spectrum of RFL for different pump power. When the pump power is above the threshold, randomly distributed spikes appear in the RFL spectrum. **b** Output power of the RFL versus pump power

The relationship between the RS and output characteristics of RS-based RFL has been analyzed theoretically based on the RS-phase-variation model above. In order to further confirm our conclusions, validation experiments were designed for the real-time and high-fidelity detection of spectral evolution, and details of the experiment setup are given in “Materials and methods”. RS-phase-variation model and rapid spectrum detection also provide theoretical and experimental methods for probing the micro-dynamic properties of RFLs as well as multi-disciplinary applications. The spectrum of RS-based RFL is shown in Fig. 2a, b shows the output RS-based RFL at the fiber end as a function of the pump power. The spectrum was measured by a high-resolution spectrometer with a resolution of 0.16 pm. To prevent excessive power from damaging the spectrometer, a 1:99 coupler was employed. Specifically, the 1% end was connected to the spectrometer for spectral measurements, and the 99% end was connected to the powermeter for optical power measurements, resulting in a 20 dB difference between the actual power level and the power reading in OSA. When the pump power is below the threshold (which is 1.3 W), it is primarily spontaneous Raman scattering. When the pump power is above the threshold, the 10 dB linewidth of the RS-based RFL spectrum is 4 GHz. Due to the stochasticity and incoherence of the RS, the spectrum of the RFL exhibits characteristics of randomly distributed peaks.

**Fig. 3:**
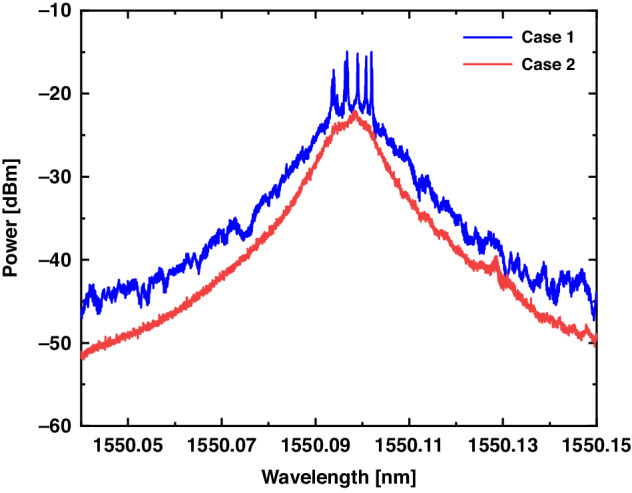
The experimental RS-based RFL spectrum of different RS phase fluctuation: Case 1: small fluctuation of RS phase with the random spikes; Case 2: large fluctuation of RS phase without the random spikes

Two experiments are demonstrated with different RS phase fluctuations. In Case 1, the optical fiber of the RS-based RFL is in a stable state, in which the degree of fluctuation of the RS phase is small (as shown in Fig. S4), and the spectrum of the RS-based RFL is shown as the blue curve in Fig. 3; otherwise, in Case 2, significant external action is applied to the optical fiber of the RS-based RFL, in which the optical range between two points of RS will be changed and leading to a larger degree of RS phase fluctuation(as shown in Fig. S5), and the spectrum is shown as the red curve in Fig. 3. Experimentally and theoretically, it is proved that when the RS phase fluctuation degree is small, a stable, coherent feedback cavity can be formed in the fiber, and the random longitudinal mode modes are excited in the RFL (which are manifested as randomly distributed spikes on the spectrum); and when the RS phase fluctuation degree is large, the randomly distributed spikes of the spectrum disappear.

### Theoretical prediction and experimental observation for photonic phase transition

**Fig. 4:**
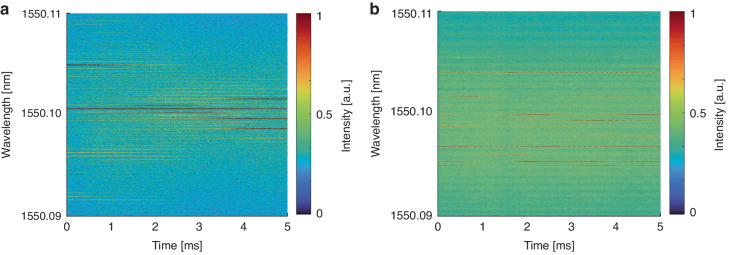
Temporal spectrogram of RFL with randomly distributed spikes. **a** Simulation. **b** Experiment

In the experiments, the real-time high-fidelity detection of spectral evolution is realized by frequency beating operation of RS-based RFL coupled with the narrow linewidth tunable laser (NLL) as well as time-frequency domain conversion based on short-time Fourier transform^44,45^. Then, to meet the bandwidth requirements and obtain more information on the spectrum, the frequency difference between NLL and the RS-based RFL was set to 9 GHz. Due to the narrow bandwidth of the randomly distributed spikes in the spectrum and the need for acquiring a large number of replicas within a short time window, high temporal and frequency precision in spectral detection is required in experiments to accurately observe photon phase transition. The evolution of the spectrum within a 5 ms interval is illustrated in Fig. 4, and the temporal resolution and wavelength resolution are 1 $\mu s$ and 2 ${MHz}$, respectively. Figure 4a shows the simulated temporal spectrogram and Fig. 4b is the experimental result correspondingly. From the temporal spectrogram, it can be observed that the intensity of randomly distributed spikes on the spectrum is not static but fluctuates over time.

The correlation among the RS-based RFL’s wavelength can be explored through Pearson’s correlation coefficient involving the statistical covariance measure between intensity fluctuations at wavelengths $\left({\lambda }_{1},{\lambda }_{2}\right)$ in the same spectrum,

1 $$\rho \left({\lambda }_{1},{\lambda }_{2}\right)=\mathrm{cov}\left({I}_{{\lambda }_{1},}{I}_{{\lambda }_{2}}\right)/\left({\sigma }_{{\lambda }_{1}},{\sigma }_{{\lambda }_{1}}\right)$$

**Fig. 5:**
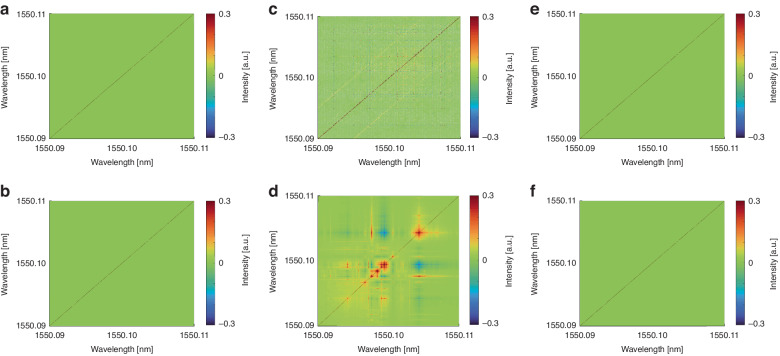
Matrix of $\rho$ values for RFL in different state. **a**, **b** Below the threshold. The different RFL modes in the frequency domain are mutually independent, $\rho =0$; **c**–**f** Above the threshold. **c**, **d** The randomly distributed spikes in the RFL spectrum exhibit correlation, while the remaining spectral components are mutually independent; **e**. **f** The photon phase variation will compromise the correlation between the random longitudinal modes; **a**, **c**, **e** Simulation. **b**, **d**, **f** Experiment

Figure 5 shows the matrix of $\rho$ values. The figures above show the simulation results, and the figures below show the corresponding experimental results. Figure 5a, b illustrates the matrix of $\rho$ values below the threshold; Fig. 5c, d presents the matrix of $\rho$ values above the threshold with stable photon phase; Fig. 5e, f depict the matrix of $\rho$ values above the threshold with large fluctuation of photon phase. When the pump power is below the threshold, there is no mutual correlation among the spectral wavelengths. However, when the pump power exceeds the threshold, the spectral correlation of RS-based RFL exhibits mode-asymmetry. Specifically, this is attributed to gain competition and sharing, leading to nonlinear interactions among randomly distributed spikes and resulting in spectral correlation, while the remaining components of the spectrum remain uncorrelated. Due to these mutual interactions among the randomly distributed spikes, the intensity of the narrowband components exhibits fluctuations. As shown in Fig. 5e, f, violent fluctuations of the photon phase will disrupt the interactions between different longitudinal modes.

In order to investigate the photon phase transition based on spectral intensity fluctuations, spectral data below and above the threshold were separately selected. For the experiment, 1000 spectra constitute a set of replicas. It is worth noting that each dataset has a temporal span of 5 ms, within which the experimental conditions remain consistent. So, each dataset can be regarded as a set of replicas. For the simulation, 1000 spectra were chosen as a set of replicas which are under the same simulation conditions. The intensity fluctuations within each set of replicates are as follows:

2 $${\Delta }_{\alpha }\left(k\right)={I}_{\alpha }\left(k\right)-\bar{I}\left(k\right)$$

where the $\bar{I}\left(k\right)$ is the average over replicas of each wavelength intensity. The overlap parameter of the replicates is defined as

3 $${q}_{\alpha \beta }=\frac{{\sum }_{k=1}^{N}{\Delta }_{\alpha }\left(k\right){\Delta }_{\beta }\left(k\right)}{\sqrt{{\sum }_{k=1}^{N}{\Delta }_{\alpha }^{2}\left(k\right)}\sqrt{{\sum }_{k=1}^{N}{\Delta }_{\alpha }^{2}\left(k\right)}}$$

Based on the cross-correlation of the spectrum, the set of values of ${q}_{\alpha \beta }$ is calculated and the probability distribution P(q) of ${q}_{\alpha \beta }$ values is determined for several pump powers.

**Fig. 6:**
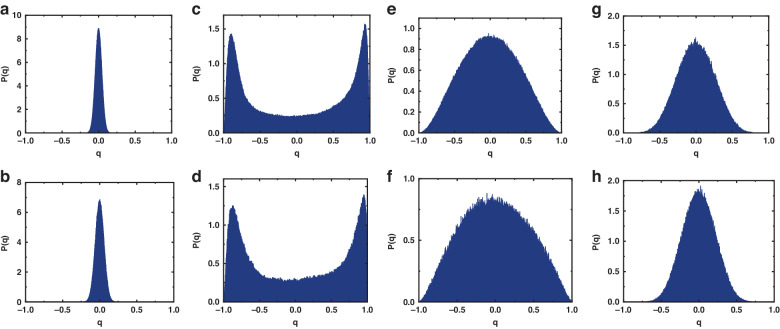
Distribution function of the overlap q at different pump powers. **a**, **b** below the threshold, which means RS-based RFL is in paramagnetic state. **c**–**h** above the threshold: **c**, **d** randomly distributed spikes of RS-based RFL, which means the RSB has set in; **e**, **f** remaining spectral components (the slowing varying spectral profile over wavelength and time) of RS-based RFL, which mean the remain spectral components is still in paramagnetic state; **g**, **h** the photon phase variation disrupts the spin-glass state. Different parts of the spectrum exhibit different states consistent with their corresponding spectral correlations, which shows mode asymmetric, and photon phase variation can be analogized to “high temperature”. **a**, **c**, **e**, **g** Simulation. **b**, **d**, **f**, **h** Experiment

Figure 6 shows the P(q) for different pump powers. The figures above show the simulation results, and the figures below show the corresponding experimental results. As shown in Fig. 6a, b, when pump power is below threshold, P(q) is centered around *q* = 0. In this case, we identify the maximum of the distribution P(q) as ${q}_{\max }=0$, which means that each mode of the RS-based RFL is independent and essentially does not interact with others at the photonic paramagnetic phase. Remarkably, when the pump power exceeds the threshold, corresponding to the mode-asymmetry in the spectral correlation, the photon phase transition of RS-based RFL also exhibits mode-asymmetry correspondingly. Specifically, different components of the RS-based RFL exhibit entirely distinct characteristics, which is a surprising new discovery. Specifically, when the pump power surpasses the threshold, the RS-based RFL emits random longitudinal modes based on coherent feedback, appearing as randomly distributed peaks in the spectrum. Due to gain competition and gain sharing, these random longitudinal mode patterns are no longer independent; instead, they exhibit strong mutual relationships in the frequency domain. By extracting the randomly distributed peaks in the RS-based RFL spectrum as a set of replicas, the values of q are calculated. As illustrated in Fig. 6c, d, we find ${q}_{\max }=1$ (in absolute value), indicating that RSB has set in. However, the remaining components of the spectrum remains in the paramagnetic state, maintaining mutual independence in the frequency domain. By selecting the same data points from the remaining part of the spectrum as a set of replicas, now we find ${q}_{\max }=0$, as shown in Fig. 6e, f.

Experimental and simulation results demonstrate the mode-asymmetry in the phase transition of the RS-based RFL: When the energy landscape of the RS-based RFL changes, different longitudinal modes of RS-based RFL undergo different phase transition processes due to different degrees of nonlinear interactions. Specifically, for random longitudinal modes excited by coherent RS, strong nonlinear interactions lead to a photon phase transition from the paramagnetic phase to the spin glass phase; in contrast, due to weak nonlinear interaction, modes based on incoherent RS maintain the paramagnetic phase. Additionally, as illustrated in Fig. 6g, h, when the photon phase of the RS-based RFL undergoes an intense fluctuation, the disappearance of the interactions among the random longitudinal modes leads to the overall alignment of the RFL in a paramagnetic state, akin to conditions with elevated temperatures.

**Fig. 7:**
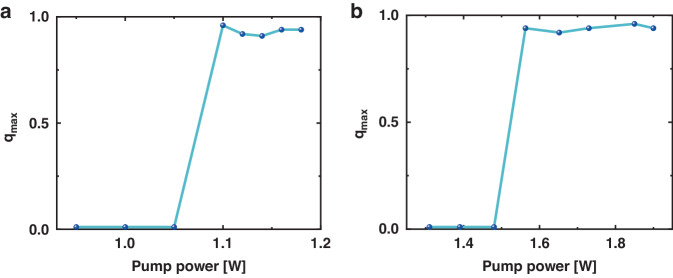
The change of ${q}_{\max }$ showing a phase transition in random longitudinal modes of RFL as pump power increases. **a** Simulation. **b** Experiment

The relationship between ${q}_{\max }$ and the pump power is depicted in Fig. 7, and the experimental findings align with those obtained through simulation, exhibiting a congruent trend. As the pump power transitions from below the threshold to above it, ${q}_{\max }$ changed from 0 to 1, which means a phase transition.

**Fig. 8:**
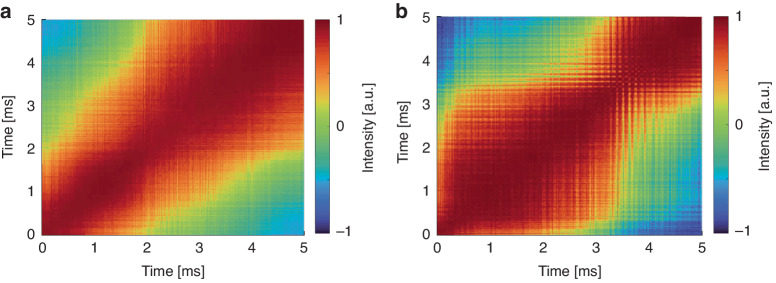
Temporal correlation coefficient of the randomly distributed spikes of RS-based RFL, which shows high degree of correlation. **a** Simulation. **b** Experiment

Furthermore, the temporal correlation within narrowband components of the RS-based RFL was also investigated. Figure 8 shows the matrix of $\rho$ values for the temporal correlation. The left-hand side shows the simulation results, and the right-hand side shows the corresponding experimental results. As shown in Fig. 8, the narrowband components of the laser emission spectrum exhibit a high degree of temporal correlation. Through the manipulation of spectral randomly distributed spikes, such as the introduction of frequency shifts within the optical cavity, the correlation characteristics of the RS-based RFL can be controlled. The experimental results match well with the simulation results.

## Discussion

As a promising approach for investigating complex disordered systems, we reported the in-depth study of the intrinsic mechanisms of the RSB in the RS-based RFL, both theoretically and experimentally, for the first time. Using a $\Phi$-OTDR with proprietary technologies, the RS phase fluctuations over time with unprecedented precision are measured, from which an RS-phase-variation model of RS-based RFL is proposed. Notably, from the model and the high-precision spectral measurement, we not only revealed the role of the photon phase variation in phase transition but also observed the mode-asymmetric of the phase transition in RS-based RFL: specifically, the RSB is observed at the randomly distributed spikes (corresponding to coherent feedback-induced random longitudinal modes), and these modes display strong interactions in both the time and frequency domains; but the remaining spectral components (the slowing varying spectral profile over wavelength and time) remain in a paramagnetic state, exhibiting weak interactions.

The spin glass theory with RSB phenomenon, a challenging topic in condensed matter physics, involves complex arrangements and interactions of spin degrees of freedom. In this work, due to the high disorder of RS, RS-based RFL provides an ideal theoretical and experimental platform for the study of RSB spin glass theory. The study of this theory can not only promote the progress of condensed matter physics^46^ and deepen the understanding of the behavior of complex spin systems, but also help to resolve the quantum phase transition mechanism.

## Materials and methods

### Theoretical model

The RS phase variation model is shown below:

4 $$\frac{\partial {u}_{p}^{\pm }}{\partial z}\mp \frac{1}{{v}_{{gs}}}\frac{\partial {u}_{p}^{\pm }}{\partial t}\pm i\frac{{\beta }_{2p}}{2}\frac{{{\partial }^{2}u}_{p}^{\pm }}{\partial {t}^{2}}\pm \frac{{\alpha }_{p}}{2}{u}_{p}^{\pm }=\pm i{\gamma }_{p}{\left|{u}_{p}^{\pm }\right|}^{2}{u}_{p}^{\pm }\mp \frac{{g}_{p}\left(\omega \right)}{2}\left(\left\langle {\left|{u}_{s}^{\pm }\right|}^{2}\right\rangle +\left\langle {\left|{u}_{s}^{\mp }\right|}^{2}\right\rangle \right){u}_{p}^{\pm }$$

5 $$\begin{array}{ll}\frac{\partial {u}_{s}^{\pm }}{\partial z}\pm i\frac{{\beta }_{2s}}{2}\frac{{{\partial }^{2}u}_{s}^{\pm }}{\partial {t}^{2}}\pm \frac{{\alpha }_{s}}{2}{u}_{s}^{\pm }\mp \frac{\varepsilon \left(\omega ,t\right)}{2}{u}_{s}^{\pm }\\=\pm i{\gamma }_{s}{\left|{u}_{s}^{\pm }\right|}^{2}{u}_{s}^{\pm }\pm \frac{{g}_{s}\left(\omega \right)}{2}\left(\left\langle {\left|{u}_{p}^{\pm }\right|}^{2}\right\rangle +\left\langle {\left|{u}_{p}^{\mp }\right|}^{2}\right\rangle \right){u}_{s}^{\pm }\end{array}$$

where subindexes ′*p*′ and ′*s*′ represent the pump and Stokes waves respectively; ′+′ and ′−′ correspond to forward and backward light; $u$ is the envelope of the optical field; ${v}_{{gs}}$ is the group velocity difference arising from the wavelength discrepancy between the pump and Stokes waves; $\omega$ is the angular frequency of lightwave; $\alpha ,\gamma ,{\beta }_{2}$ and $g$ are the linear fiber loss, Kerr coefficient, second-order dispersion, and Raman gain respectively, and $g$ are related to frequency in this simulation; $\varepsilon$ is RS, whose intensity and phase are related to frequency and time; since the degree of time-dependent RS phase fluctuation determines the output state of RFL, in order to achieve accurate simulation of RFL, the phase of RS is precisely measured by a $\Phi$-OTDR with proprietary technologies.

The boundary conditions can be described as:

6 $$\begin{array}{ll}{P}_{p}^{+}\left(0,\omega ,t\right)={P}_{{in}}\left(\omega \right){T}_{{L}_{p}}+{R}_{{L}_{p}}\left(\omega \right){P}_{p}^{-}\left(0,\omega ,t\right),{P}_{p}^{-}\left(L,\omega ,t\right)\\\qquad\qquad\quad=\,{R}_{{R}_{p}}\left(\omega \right){P}_{p}^{+}\left(L,\omega ,t\right)\end{array}$$

7 $${P}_{s}^{+}\left(0,\omega ,t\right)={R}_{{L}_{s}}\left(\omega \right){P}_{s}^{-}\left(0,\omega ,t\right),{P}_{s}^{-}\left(L,\omega ,t\right)={R}_{{R}_{s}}\left(\omega \right){P}_{s}^{-}\left(L,\omega ,t\right)$$

where ${R}_{L}$ and ${R}_{R}$ are the reflection spectrum of the FBG and the fiber respectively; ${P}_{{in}}$ denotes the input pump power; $L$ is fiber length, which is 15 km. The parameter values set in this simulation are shown in Table 1, in which $\bar{\varepsilon }$ is average intensity of RS.

**Table 1:** Parameters set in the simulation (see text for definitions)

Parameter	Pump	Stokes
${\lambda}({\rm{nm}})$	1455	1550
${v}_{g}$($m/s$)	2.0504 × 10^8^	2.0497 × 10^8^
$\alpha$(${dB}/{km}$)	0.24	0.2
$g$(${m}^{-1}{W}^{-1}$)	4.14 × 10^−4^	–
$\gamma$(${m}^{-1}{W}^{-1}$)	0.0017	0.0014
$\bar{\varepsilon }$(${m}^{-1}$)	0.6 × 10^−7^	0.45 × 10^−7^
${\beta }_{2}$(${s}^{2}/m$)	−1.7324 × 10^−26^	−2.7927 × 10^−26^
${R}_{L}$	4 × 10^−5^	0.99
${R}_{R}$	4 × 10^−5^	4 × 10^−5^

#### Experimental setup

**Fig. 9:**
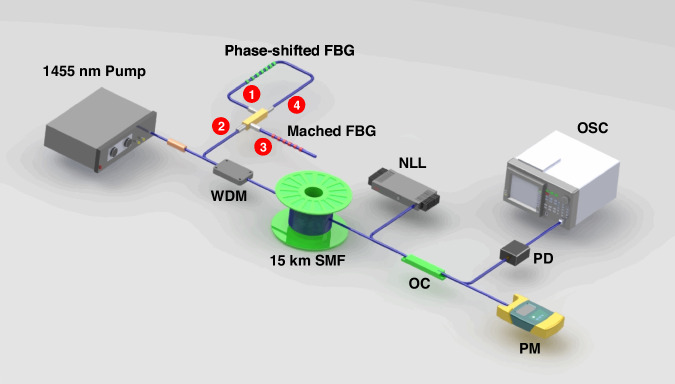
Experiment setup for rapid spectrum detection. WDM wavelength division multiplexer, SMF single model fiber, NLL narrow linewidth tunable laser, OC optical coupler, PD photodetector, OSC oscilloscope, PM power meter

The setup of the experiment is shown in Fig. 9, which is consistent with the simulation. The 1455 nm pump source injects into a 15 km single mode fiber through a 1455/1550 nm wavelength division multiplexer and the 1550 nm port is connected to the feedback. It is worthy to note that limited by the bandwidth of photodetector, the transmission peak of the phase-shift FBG is chosen as the feedback of the RFL, which is 0.02 nm. The generated RS-based RFL outputs at the end of the fiber and in order to achieve rapid and high-precision spectral detection, NLL with the linewidth <100 Hz is used in conjunction with the generated RS-based RFL for beat-frequency operation in the experiment^44,45^, and the power meter is employed to monitor the laser power, preventing potential damage to the detector caused by excessive power. Time-domain data is collected using a 40 GHz bandwidth detector and a 16 GHz bandwidth oscilloscope, and the data acquisition duration for each dataset is 5 ms.

### Supplementary information

Supplementary information for Replica symmetry breaking in 1D Rayleigh scattering system: theory and validations

## Data Availability

The data are available from the corresponding authors upon reasonable request.
