# Supplementary material for: Replica symmetry breaking in 1D Rayleigh scattering system: theory and validations
Source: Light Sci Appl. 2024 Jul 2;13:151. doi: 10.1038/s41377-024-01475-5 (PMC11219881; doi:10.1038/s41377-024-01475-5)
Supplement: Supplementary file 1 — Supplementary information for Replica symmetry breaking in 1D Rayleigh scattering system: theory and validations [file 41377_2024_1475_MOESM1_ESM.docx]

Supplementary information for

Replica symmetry breaking in 1D Rayleigh scattering system: theory and validations

# Yifei Qi1, Longqun Ni1, Zhenyu Ye1, Jiaojiao Zhang1, Xingyu Bao1, Pan Wang1, Yunjiang Rao1, Ernesto P. Raposo2,*, Anderson S. L. Gomes3,* and Zinan Wang1,*

*1 Key Lab of Optical Fiber Sensing & Communications, University of Electronic Science and Technology of China (UESTC), Chengdu, China*

*2Laborato´rio de F´ısica Teo´rica e Computacional, Departamento de F´ısica, Universidade Federal de Pernambuco, 50670-901 Recife, Pernambuco, Brazil*

*3Departamento de F´ısica, Universidade Federal de Pernambuco, Recife, Pernambuco, Brazil*

[ernesto.raposo@ufpe.br](mailto:ernesto.raposo@ufpe.br)

[andersonslgomes@gmail.com](mailto:andersonslgomes@gmail.com)

[znwang@uestc.edu.cn](mailto:znwang@uestc.edu.cn)

**Supplementary Information: The measurement of RS phase fluctuations based on a** $\boldsymbol{\Phi}$**-OTDR with proprietary technologies**

The experimental setup of $\Phi$-OTDR is shown in Fig. 1. A narrow linewidth laser is split into two branches using a 90:10 coupler, where 90% constitutes the probe signal branch and 10% forms the local oscillation branch. The signal light is modulated with the IQ modulator and the modulated signal is a 50 MHz-1550 MHz chirp pulse with a repetition period of 60 μs and a pulse width of 1 μs. Compared to traditional pulse signals, chirped pulses are able to surpass the conventional trade-off between pulse width and spatial resolution, thereby achieving higher signal-to-noise ratio and spatial resolution in the system. Following amplification via an EDFA and the removal of ASE noise introduced by a FBG, the chirp pulse is injected into a 1 km SMF. Within the fiber, non-uniformities in the refractive index cause the scattering of a portion of the chirp pulse, leading to the generation of RS light. At the receiver, the backward RS light is heterodyned with the local oscillating light, employing the polarization controller (PC) of the local oscillating branch to mitigate polarization-induced effects on system performance. The receiver's signal is sampled at a rate of 5 GSa s^-1^ with a sampling time of 16 ms.


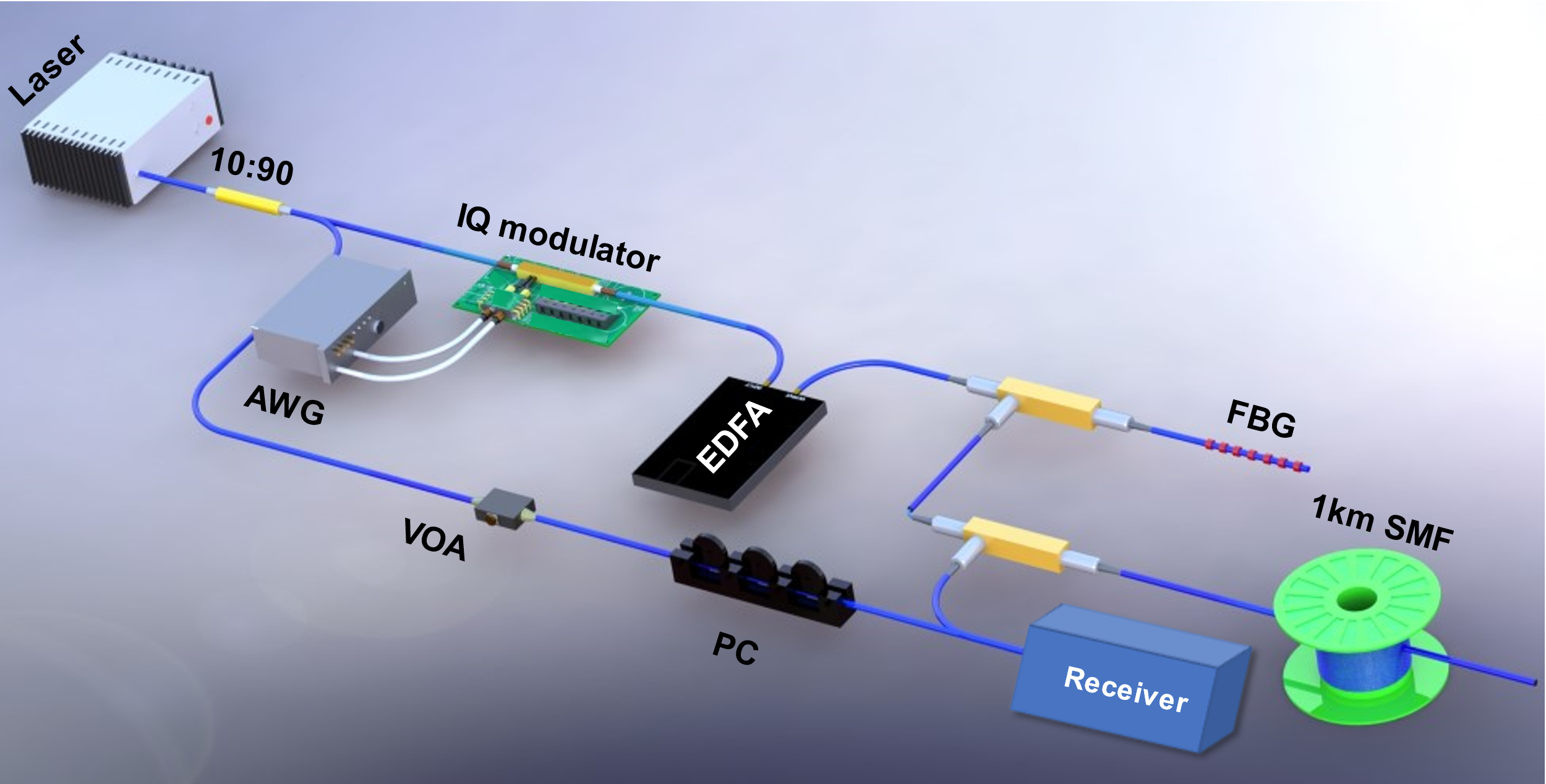


**Figure S1** The experimental setup of $\Phi$-OTDR: AWG, arbitrary waveform generator; EDFA, Erbium-doped fiber amplifier; VOA, variable optical attenuator; PC, polarization controller.

Firstly, the RS reflection spectrum is measured and shows randomly distributed spikes, which is shown in Fig. S2. When the fiber is in the experimental environment, the RS reflection spectra at different moments are shown in Fig. S3a, and the randomly distributed spikes on the spectrum remain stable. However, when an external action is applied to the fiber, the RS reflectance spectra at different moments show different spectral characteristics. Disturbances can cause slight variations in the length of optical fibers, resulting in slight shifts in the frequency and phase of Rayleigh scattered light, manifesting themselves as variations in peak positions and intensities in the spectra, thereby affecting their degree of overlap, illustrated in Fig. S3b.


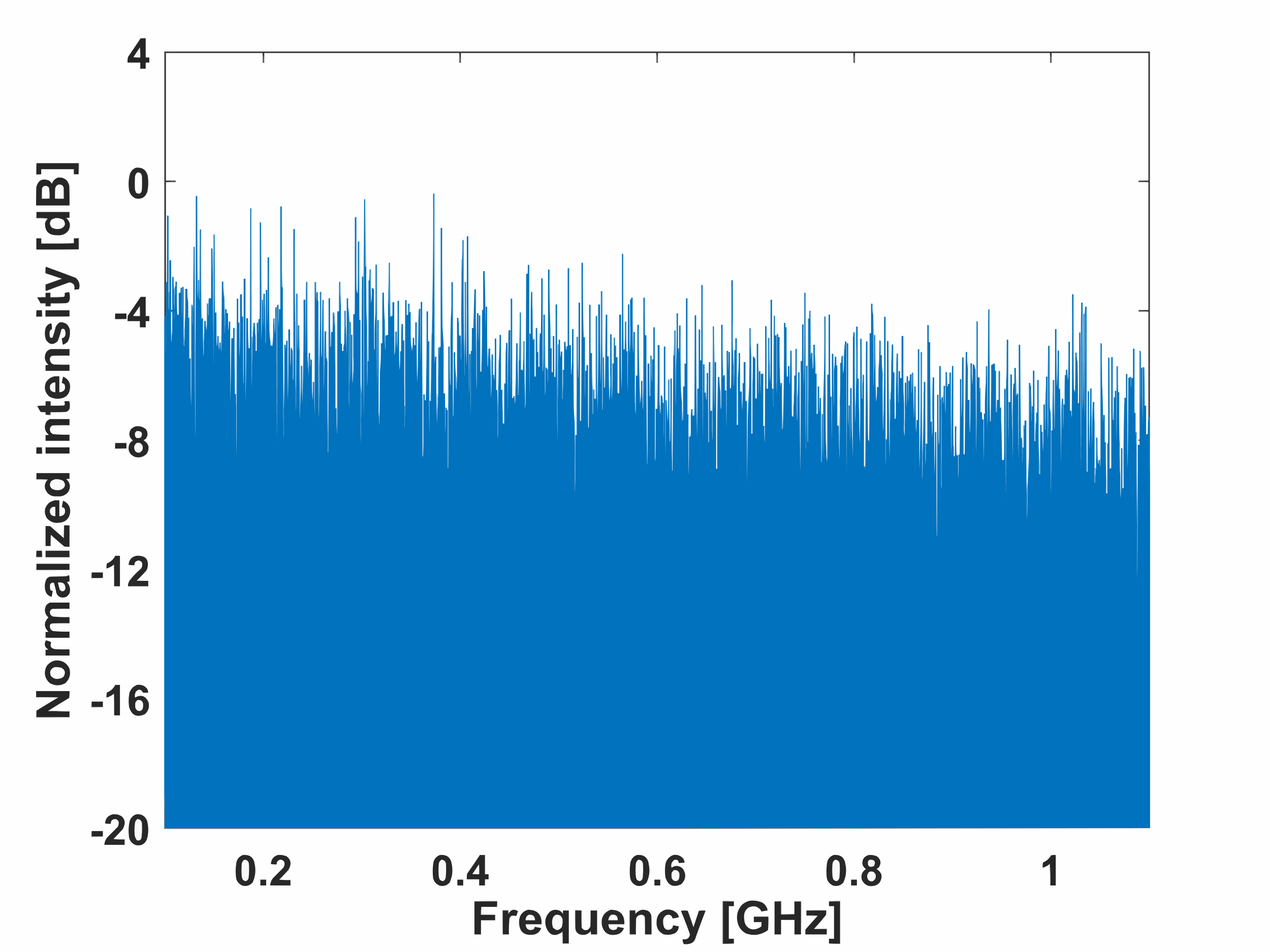


**Figure S2** RS reflectance spectrum, which exhibits randomly distributed spikes. The coherent feedback formed between these spikes leads to the appearance of randomly distributed spikes in the RFL.


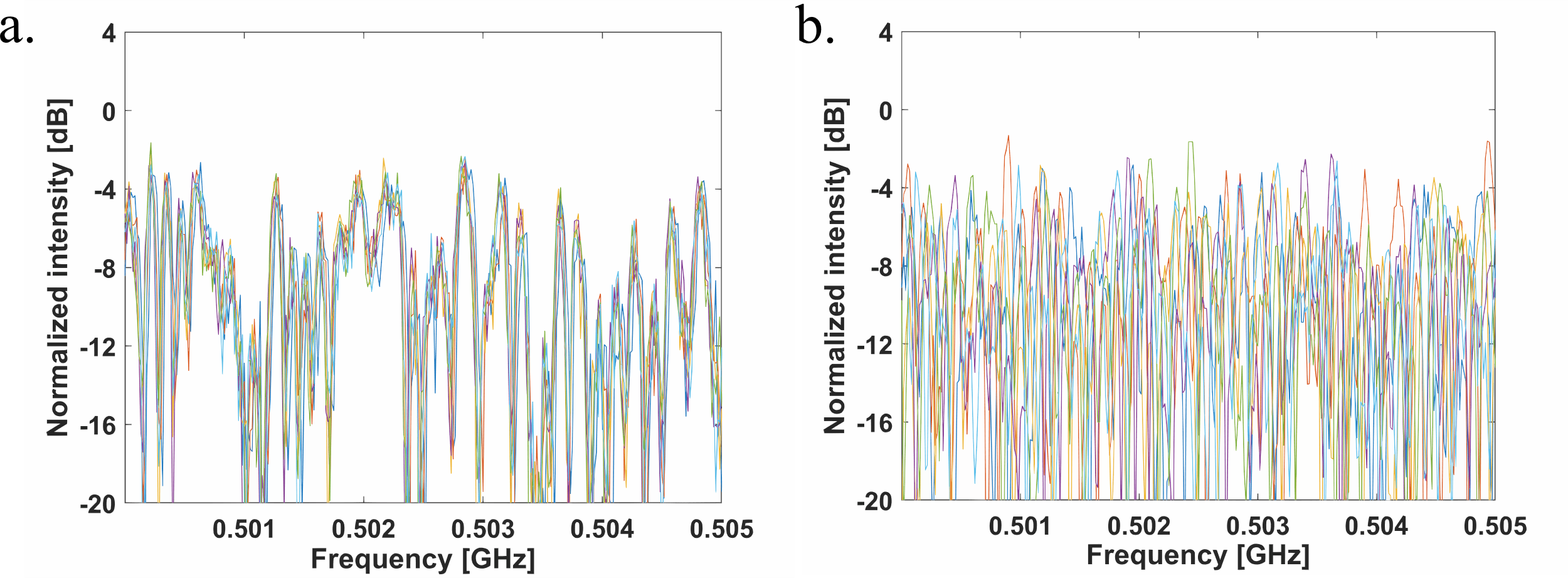


**Figure S3 a.** The RS reflection spectrum at different time with only environmental noise: the randomly distributed spikes of the RS reflectance spectra remain stable; **b.** The RS reflection spectrum at different time with external fierce perturbation: RS reflectance spectra at different moments exhibit different spectral properties.

Then, the RS phase of optical fiber in different states is measured. The frequency shift between different traces is computed using the LMS algorithm ^[39]^, and this frequency shift is converted into phase variations based on their relationship. The phase of the demodulated signal is affected by system noise, which includes the environmental noise, the noise of the receiver, and ASE noise. However, considering the 1 km fiber length and a sampling time of 16 ms, the impact of laser frequency drift can be disregarded. The phase fluctuations of RS caused by environmental noise are first measured, as shown in Fig. S3, where each curve is the RS phase curve at different time with a time interval of 60 μs. The overall RS phase fluctuation is less than 0.4 rad, and the RS-phase-variation model will be calibrated based on this measurement.


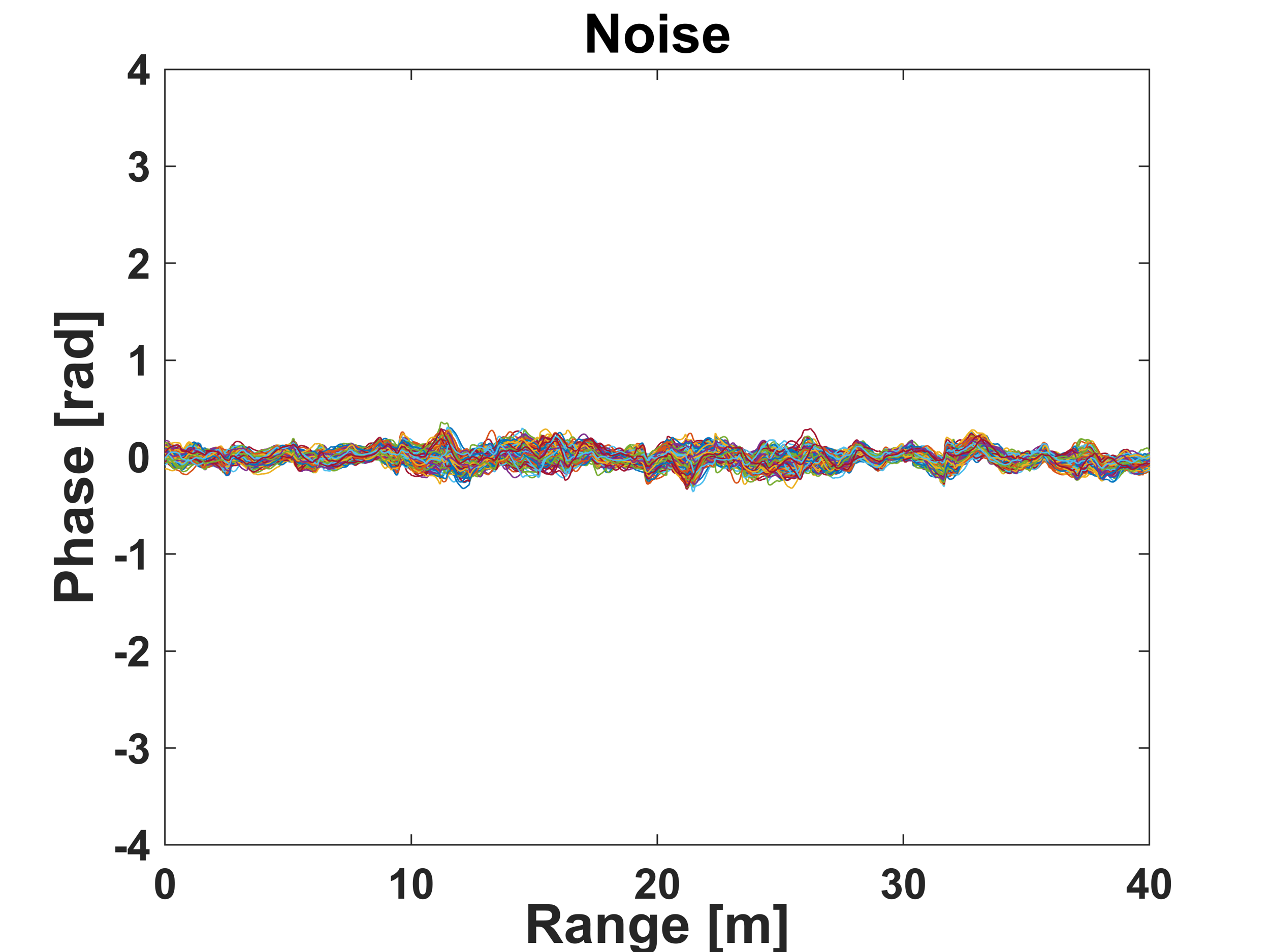


**Figure S4** The phase of the RS fluctuations caused by environmental noise: the time interval for each curve is 60 μs, and the total time is 16 ms, which is consistent with the simulation settings, and the overall RS phase fluctuation range is less than 0.4 rad.

Subsequently, the RS phase fluctuations were measured for the case of applying perturbation to the fiber, illustrated in Fig. S5, which is approximately 5 rad. The dynamic changes of RS phase caused by the disturbances are depicted in Fig. S6, where the RS phase fluctuates randomly between -2.5 rad and 2.5 rad. Based on this measurement, it is shown that the RS-phase-variation model can be verified by studying the RS-based RFL output characteristics of the fiber being perturbed.


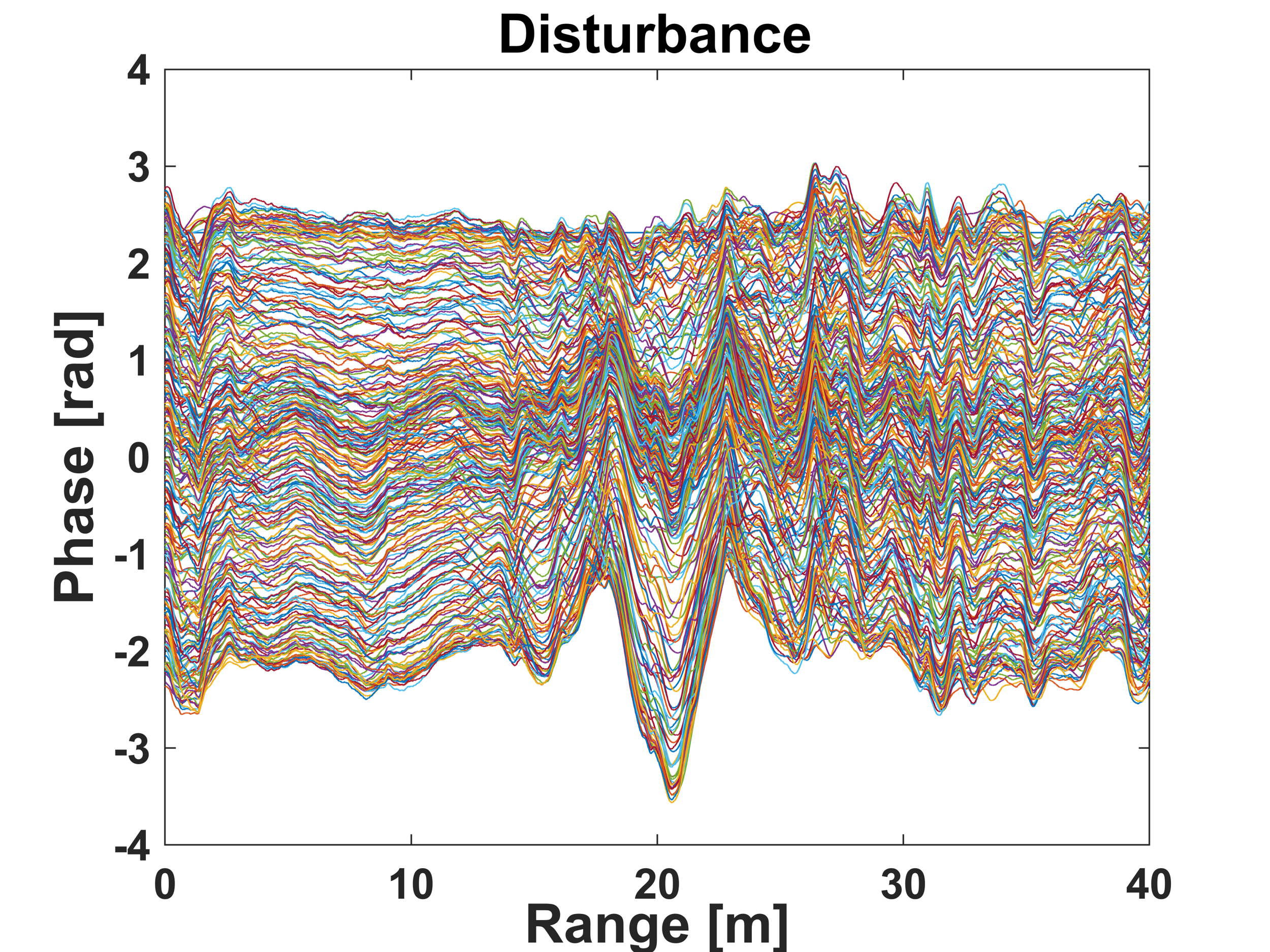


**Figure S5** The fluctuation range (about 5 rad) of the RS phase caused by external action：the time interval for each curve is 60 μs, and the total time is 16 ms, which is consistent with the simulation settings.


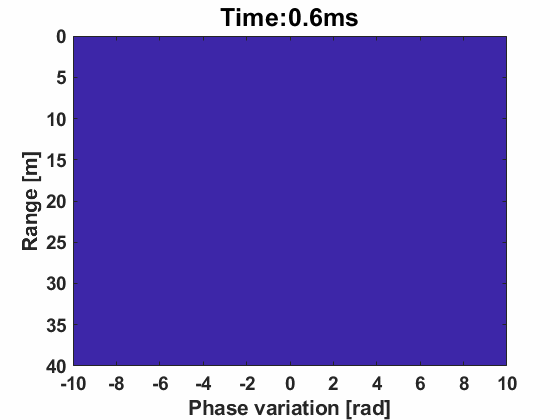


**Figure S6** Dynamic change in RS phase. By applying an external action, the phase of RS fluctuates randomly in the range of -2.5 rad to 2.5 rad. (This animation requires Word 2021 or a higher version.)
